# Highly elevated sepsis biomarkers in advanced cholangiocarcinoma without sepsis: A case report and literature review

**DOI:** 10.1097/MD.0000000000042115

**Published:** 2025-05-23

**Authors:** Bianca Karnuth, Almut Brundert, Claus Langer, Thomas Masetto, Christian Müller, Maximilian Jüdt, Yuriko Stiegler, Hugo Stiegler, Christoph Peter, Matthias Grimmler

**Affiliations:** a Medizinisches Versorgungszentrum für Labormedizin und Mikrobiologie Ruhr GmbH, Essen, Germany; b Department of Medical Oncology, Evangelische Kliniken Essen-Mitte gGmbH, Essen, Germany; c Institute of Molecular Medicine I, Medical Faculty, Heinrich Heine University Düsseldorf, Düsseldorf, Germany; d DiaSys Diagnostic Systems GmbH, Holzheim, Germany; e Institute for Biomolecular Research, Hochschule Fresenius gGmbH, University of Applied Sciences, Idstein, Germany; f DiaServe Laboratories GmbH, Iffeldorf, Germany.

**Keywords:** biomarker, case report, cholangiocarcinoma, liver, procalcitonin, sepsis

## Abstract

**Rationale:**

Inflammatory markers such as C-reactive protein (CRP) and interleukin-6 (IL-6) are often elevated in liver cancer, making it difficult to monitor for bacterial infection. Hence, it is tempting to use more bacterial-specific sepsis markers such as procalcitonin (PCT) during immunosuppressive chemotherapy. This case study highlights the challenges of interpreting clinical chemistry sepsis biomarkers in patients with advanced cholangiocarcinoma (CCA).

**Patient concerns:**

A 55-year-old man presented with a liver mass on routine ultrasonography. MRI and CT showed multiple liver and bone metastases. The immunohistochemistry findings were consistent with an adenocarcinoma of the pancreaticobiliary system. After the diagnosis of primary hepatic CCA (NTM stage IV; FGFR2-SHROOM3 translocation) and 14 months of chemotherapy, the patient developed progressive liver lesions and new lung metastases.

**Diagnoses and interventions:**

During the last chemotherapy, PCT was highly elevated (>100 ng/mL), usually observed in severe sepsis or septic shock, whereas CRP was moderately elevated (<50 mg/L). The patient had mild leukopenia but no fever, systemic infection or septic shock. Blood and urine cultures were negative.

**Outcomes:**

After referral to best supportive care, the patient died of liver failure. Retrospective blood analysis revealed high levels of soluble CD14 subtype, a bacterial sepsis marker known as presepsin. Calcitonin and IL-6 levels were above normal, consistent with advanced CCA, but not with a PCT/calcitonin-secreting tumor or systemic inflammation.

**Lessons:**

Oncologists are aware that CRP and IL-6 values can be elevated in liver cancer. Here, we further demonstrate that highly elevated, septic shock-like PCT values can occur even in the absence of bacterial sepsis. In addition, presepsin may be elevated, although mechanistically unrelated to PCT. Therefore, sepsis markers should be interpreted with caution and in the clinical context, not only in patients with neuroendocrine or hepatocellular carcinoma, which are known to secrete PCT and calcitonin, but also in patients with advanced CCA.

## 1. Introduction

Cholangiocarcinomas (CCAs) are highly invasive adenocarcinomas derived from the biliary epithelium and represent the second most common type of primary liver cancer after hepatocellular carcinoma (HCC).^[1–3]^ Patients with intrahepatic CCA arising from the peripheral bile ducts often present with a liver mass on routine sonography.^[2]^ Most patients are asymptomatic in the early stages, and the majority of tumors are advanced and unresectable at diagnosis.^[1–3]^ Mortality from CCA has increased worldwide in recent decades, and first-line chemotherapy has shown limited efficacy, with a median overall survival of 1 year.^[2–5]^

Procalcitonin (PCT) is the 116-amino acid precursor of calcitonin, a hormone produced primarily by neuroendocrine cells, particularly in the liver and thyroid.^[6,7]^ In healthy individuals, PCT is virtually undetectable in the circulation (<0.1 ng/mL). Serum PCT is a well-established biomarker for distinguishing bacterial from nonbacterial infections and is widely used for therapeutic decision-making and antibiotic stewardship.^[6,8,9]^ The highest PCT levels are observed in patients with systemic bacterial infection and sepsis, which is defined as “life-threatening organ dysfunction caused by a dysregulated host response to infection,” or septic shock, which can be clinically identified by the requirement for vasopressors to increase the mean arterial blood pressure despite fluid resuscitation.^[7,8]^

Antimicrobial therapy is strongly recommended for patients with PCT levels > 0.5 ng/mL.^[7,9]^ According to a recent meta-analysis, this most commonly used sepsis cutoff provides 76% sensitivity and 69% specificity.^[10]^ In general, PCT and C-reactive protein (CRP) are the 2 biomarkers with the highest consensus recommendation as sepsis biomarkers.^[11]^ However, a slightly higher PCT sepsis cutoff (1.1–1.5 ng/mL) has been proposed for metastatic solid tumors, due to the elevated basal PCT levels in these patients, especially those with neuroendocrine or hepatocellular carcinoma and multiple metastases.^[12–16]^ Soluble CD14 subtype (sCD14-ST), also known as presepsin, is considered a novel and highly specific marker of bacterial infection.^[17,18]^ Until now, there have been no reports of elevated sCD14-ST/presepsin levels in cancer patients due to nonbacterial causes.

With this case report, we aim to highlight that septic shock-like biomarker elevations can occur in patients with advanced CCA even in the absence of bacterial sepsis. Therefore, markedly elevated PCT and presepsin levels should be interpreted with caution in patients with advanced primary liver cancer and confirmed using independent diagnostic tools.

## 2. Case presentation

### 2.1. Initial diagnosis of primary hepatic CCA

**Figure 1.**
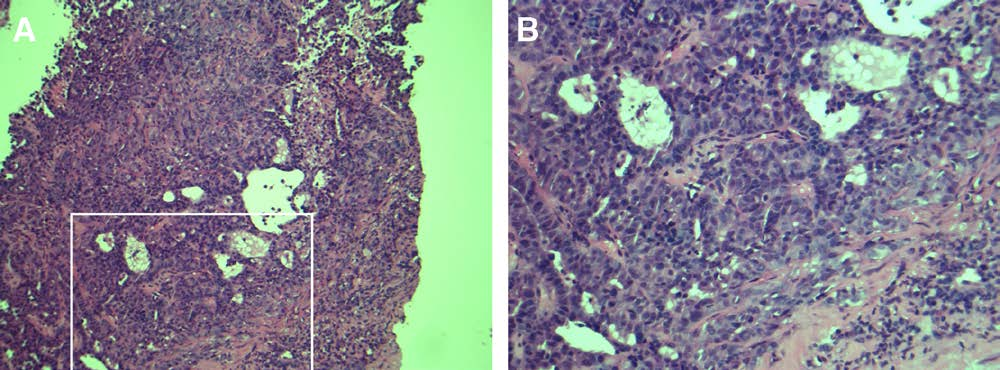
(A) H&E-stained liver biopsy showed an epithelial tumor with numerous nuclear atypia, mitoses, and marked desmoplastic stromal formation, growing partly in solid and partly in glandular association. (B) Framed section at higher magnification.

**Figure 2.**
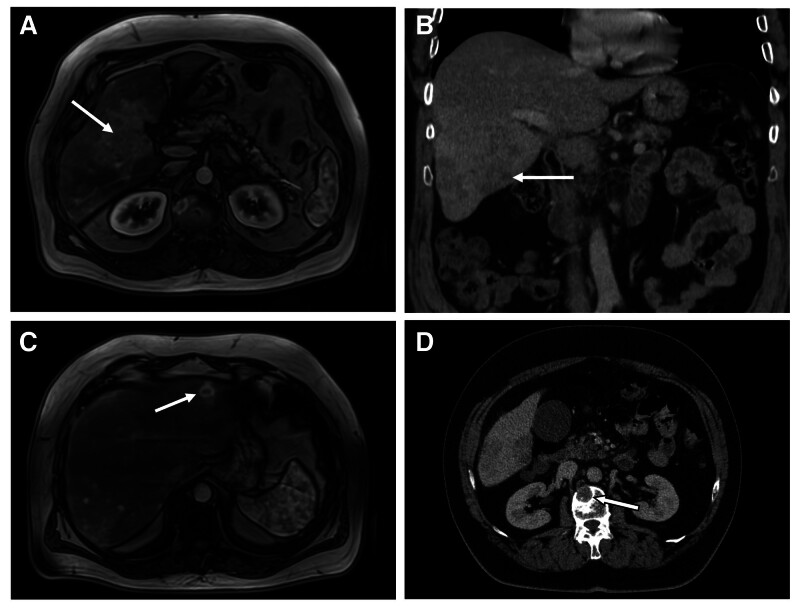
(A) Abdominal magnetic resonance imaging (MRI) showed an intrahepatic mass in the right lobe (segment IV/V). (B) Computed tomography (CT) scan of the same intrahepatic mass subsequently diagnosed as primary hepatic cholangiocarcinoma. (C) MRI revealed metastatic lesions in the left liver lobe (segment II/III). (D) CT scan showed an osteolytic bone metastasis in the lumbar spine (L2).

In October 2020, a 55-year-old man presented with an intrahepatic mass on routine sonography. The patient was obese (BMI: 34) with a history of type 2 diabetes and arterial hypertension. He worked as a dispatcher without regular contact with toxic noxae. A liver biopsy revealed a highly desmoplastic adenocarcinoma (Fig. 1). Esophagogastroduodenoscopy and colonoscopy showed no evidence of malignancy. Abdominal magnetic resonance imaging (MRI) and computed tomography (CT) showed an intrahepatic mass in the right lobe (segment IV/V; Fig. 2A/B). Diagnostic imaging revealed secondary lesions in the left liver lobe (segment II/III; Fig. 2C) and osteolytic bone metastases in the lumbar spine. Immunohistochemistry of the CT-guided liver biopsy was consistent with an adenocarcinoma of the pancreaticobiliary system: positive for mucin-1 (MUC1), but negative for MUC2, MUC5AC and prostate-specific antigen.

### 2.2. Chemotherapy and progression

Following the diagnosis of primary hepatic CCA (TNM stage IV), the patient received 3 cycles of first-line chemotherapy with cisplatin and gemcitabine and anti-osteoporosis therapy with denosumab between December 2020 and January 2021. Gastroscopy revealed multiple small gastric ulcers (Forrest III) without evidence of *Helicobacter pylori*, a type C gastritis, and Barrett esophagus without signs of intraepithelial neoplasia. Following the progression of bone metastases (Fig. 2D), the patient was switched to second-line therapy with folic acid, 5-fluorouracil, and oxaliplatin (FOLFOX). In March 2021, the patient received palliative radiotherapy to the lumbar spine (L1/L2; 30 Gy). Second-line therapy was continued until May 2021. After molecular profiling and detection of an FGFR2-SHROOM3 translocation, the therapy was switched to pemigatinib in June 2021. In November 2021, after 8 cycles of pemigatinib, a follow-up CT revealed the progression of liver lesions and newly emerging pulmonary metastases. In December 2021, the patient began chemotherapy with gemcitabine and oxaliplatin (GEMOX), which was discontinued due to thrombopenia.

A CT follow-up in January 2022 confirmed the progression of pulmonary and hepatic lesions, constant osteolytic and osteoblastic bone metastases, and nodular imbibition of the mesentery with suspicion of increasing peritoneal carcinomatosis. On 5th January, cancer antigen 19-9 was elevated, while carcinoembryonic antigen was in the normal range (Table 1). The patient had mildly impaired renal function and elevated levels of liver enzymes (Table 1; see Table S1, Supplemental Digital Content, https://links.lww.com/MD/O997, for full laboratory test results).

**Table 1:** Blood test results before and after the initiation of chemotherapy on January 12th, 2022.

Analyte	Reference range	January 18, 2022		January 17, 2022		January 12, 2022		January 05, 2022	
eGFR (mL/min/1.73)	86–107	73	–	73	–	78	–	73	–
ALP (U/mL)	40–130			243	+	314	+	328	+
AST/GOT (U/mL)	<50			69	+	118	+	119	+
ALT/GPT (U/mL)	<50			45		62	+	70	+
GGT (U/mL)	<60			519	+	520	+	576	+
CRP (mg/L)	<5	39	+	36	+	50	+	48	+
PCT (ng/mL)	<0.5	>100	++	>100	++				
WBC (10^9^/L)	3.9–10.9	2.4	‐	4.86					
RBC (cells/pL)	4.44–5.61	3.11	‐	3.34	‐				
Hb (g/dL)	13.5–16.9	9.8	‐	10.6	‐				
Platelets (10^9^/L)	166–308	85	‐	121	‐				
CA 19-9 (U/mL)	<27							200	+
CEA (ng/mL)	<3.8							1.9	

See Table S1, Supplemental Digital Content, https://links.lww.com/MD/O997, for full blood analysis.

ALP = alkaline phosphatase, ALT = alanine aminotransferase, AST = aspartate aminotransferase, CA 19-9 = carbohydrate antigen 19-9, CEA = carcinoembryonic antigen, CRP = C-reactive protein, eGFR = estimated glomerular filtration rate, GGT = gamma-glutamyl transferase, GOT = glutamic oxaloacetic transaminase, GPT = glutamic pyruvic transaminase, Hb = hemoglobin, PCT = procalcitonin, RBC = red blood cells, WBC = white blood cells.

### 2.3. Septic shock-like PCT elevation after final chemotherapy

On 12th January, the patient started chemotherapy with nanoliposomal irinotecan and 5-FU/folic acid. Five days later, the patient had a routine blood test for liver and kidney function, complete blood count, and inflammatory biomarkers (CRP and PCT). On 17th January, the serum PCT level was highly elevated above the upper detection limit of the assay (>100 ng/mL; Table 1). The extreme PCT elevation could be confirmed by additional measurement, which strongly suggested the presence of systemic bacterial infection and/or severe sepsis (Table 2; see Table S2, Supplemental Digital Content, https://links.lww.com/MD/O998, for further details). However, the septic shock-like PCT result did not correlate with the moderately elevated CRP level, which was consistent with the advanced stage of CCA and even lower than before the initiation of chemotherapy (<50 mg/L; Table 1). Blood and urine cultures remained negative for bacteria and fungi during 5 days of incubation (BACT/ALERT^®^ 3D, bioMérieux, Marcy-l’Étoile, France). Cholangitis due to biliary obstruction could be excluded, although local bacterial infections usually do not increase the serum PCT level above the 0.5 ng/mL sepsis cutoff.^[19]^ Despite moderate leukopenia and moderate thrombocytopenia (Table 1), the patient had no fever or other signs or symptoms of sepsis or septic shock.^[8]^ In February 2022, after consultation with the patient and family, chemotherapy was discontinued and the patient was transferred to best supportive care. The patient died of liver failure in March 2022.

**Table 2:** Retrospective blood analysis.

Analyte	Routine assay (Manufacturer)	Initial measurement	Additional retrospective measurements‡
Procalcitonin(PCT)	Procalcitonin FS (DiaSys Diagnostic Systems)	>100 ng/mL*	–
		>300 ng/mL†	–
	Elecsys^®^ BRAHMS PCT (Roche Diagnostics)	–	>100 ng/mL
	AFIAS PCT (Boditech)	–	>100 ng/mL
Calcitonin	Elecsys^®^ Calcitonin (Roche Diagnostics)	–	20.9 pg/mL
Presepsin (sCD14-ST)	Pathfast^TM^ Presepsin (Mitsubishi Chemicals)	–	16; 517 (±2; 309) pg/mL
Interleukin-6 (IL-6)	Elecsys^®^ IL-6 (Roche Diagnostics)	–	27.6 (±0.9) pg/mL

See Table S2, Supplemental Digital Content, https://links.lww.com/MD/O998; for further details.

* Undiluted sample exceeded the assay’s upper limit of quantification (50 ng/mL).

† Remeasurement of a diluted sample on a different analyzer.

‡ Serum samples were stored at 2 to 8 °C for 3 days and aliquoted for long-term storage at ‐80 °C.

### 2.4. Retrospective analysis of stored blood samples

To further investigate the underlying cause of the unusually high serum PCT level, we reassessed the PCT concentration using 2 additional routine assays and checked the levels of additional biomarkers by retrospectively analyzing the patient’s serum sample from 18th January (stored at ‐80 °C). Serum PCT was initially quantified using a particle-enhanced turbidimetric immunoassay – Procalcitonin ‐ FS (DiaSys – Diagnostic Systems, Germany) on 2 different clinical analyzers (Table 2). Remeasurement of a diluted sample suggested a PCT level above 300 ng/mL (Table 2). The septic shock-like PCT concentration (>100 ng/mL) on 18th January was confirmed by the Elecsys^®^ BRAHMS PCT assay (Roche Diagnostics, Germany) and the point-of-care test AFIAS PCT (Boditech Med Inc., Korea) on 2 different clinical analyzers, which rules out a measurement artifact (Table 2; see Table S2, Supplemental Digital Content, https://links.lww.com/MD/O998, for further details).

As neuroendocrine and hepatocellular carcinomas are known to secrete large amounts of calcitonin and its prohormone PCT, we analyzed the serum calcitonin level retrospectively. The calcitonin concentration was 20.9 pg/mL, which is slightly above the normal range for healthy men (10 pg/mL; Table 2). In addition, we quantified the novel and mechanistically unrelated sepsis biomarker sCD14-ST in duplicate using Pathfast^TM^ Presepsin (Mitsubishi Chemical, Japan). Interestingly, the serum concentration was relatively high (>15,000 pg/mL; Table 2), given that mean sCD14-ST/presepsin levels above 1000 pg/mL are typically observed in populations with severe sepsis or septic shock.^[18]^ Because of its central role in systemic inflammation and tumor progression, we used the Elecsys^®^ IL-6 assay (Roche Diagnostics, Germany) to measure the serum concentration of interleukin-6 (IL-6) in duplicate. In line with the increased CRP level, IL-6 was elevated above normal (27.6 ± 0.9 pg/mL; Table 2).

## 3. Discussion

Bacterial infections can be challenging to diagnose in cancer patients and are a significant cause of mortality in these patients. Blood culture is still the primary diagnostic method for bacteremia, the most common type of bacterial infection in cancer patients.^[7,20]^ The clinical usefulness of inflammation biomarkers such as PCT, CRP, and IL-6 in cancer patients to differentiate systemic bacterial infection from tumor- or therapy-related symptoms and to rule out sepsis has been widely discussed.^[20–25]^ While bacteremia and sepsis require immediate intervention, unnecessary withdrawal of chemotherapy and initiation of antibacterial therapy can result in additional side effects, reduced oncologic efficacy, and poor outcomes in patients with advanced solid tumors.^[26,27]^ In this case, a precautionary measurement of PCT was ordered after the start of nanoliposomal irinotecan/5-FU/folic acid treatment, in addition to CRP, for the early detection of any serious bacterial infection that might result from chemotherapy-induced neutropenia. As CRP is produced by hepatocytes in the liver, serum CRP was elevated above normal before the start of treatment on 12th January (50 mg/L) and even lower on 17th and 18th January (≤39 mg/L). In contrast, serum PCT was elevated > 100 ng/mL, which is more than 200-fold above the common sepsis cutoff (0.5 ng/mL) and still 100-fold above the slightly higher sepsis cutoff (1.1–1.5 ng/mL) proposed for solid tumor patients.^[12]^

### 3.1. Serum PCT elevation in patients with neuroendocrine carcinoma

Basal PCT levels above 0.5 ng/mL are well documented for non-septic patients with primary lung cancer, especially those with neuroendocrine component and multiple metastases.^[28–30]^ In addition, highly elevated serum PCT and calcitonin are frequently described for patients with neuroendocrine carcinomas (NECs) of the liver and pancreas.^[13–16]^ Most of these patients had multiple liver metastases and presented with high fever. Evidence for calcitonin and PCT secretion by the tumor accompanied by extreme calcitonin serum levels (>2000 pg/mL) was provided for 3 patients with pancreatic NEC.^[14–16]^ Although we could not test for PCT or calcitonin secretion by the tumor itself, the serum concentration of calcitonin was 20.9 pg/mL, which is above the normal range (10 pg/mL), but well below the levels reported in the NEC patients mentioned above. Calcitonin levels > 100 pg/mL are highly predictive of medullary thyroid cancer (MTC), whereas concentrations > 500 pg/mL indicate multiple MTC metastases.^[31]^ In conclusion, these results suggest that basal serum PCT levels may be elevated well above the sepsis cutoff in cancer patients, even in the absence of systemic bacterial infections. However, extreme elevation in PCT (>100 ng/mL) and calcitonin (>500 pg/mL) were observed mainly in metastatic NECs, which can secrete large amounts of PCT and calcitonin.^[13–16]^

### 3.2. Serum PCT elevation in patients with primary liver cancer and CCA

Several cell types have been suggested to contribute to the increased PCT secretion during sepsis, including leukocytes, monocytes, and neuroendocrine cells in various tissues. However, the liver is regarded as the major source of PCT during bacterial infection.^[32]^ Only a few case reports of primary liver cancer have been published so far that displayed high serum PCT levels (>10 ng/mL) without signs of bacteremia or sepsis, including 5 patients with HCC or fibrolamellar HCC (FL-HCC). See Table S3, Supplemental Digital Content, https://links.lww.com/MD/O999, for further details. To our knowledge, only 2 similar cases of CCA have been described thus far. Meegada et al reported an 80-year-old male patient with intrahepatic CCA and a history of chronic kidney disease (CKD stage III) and acute kidney injury who presented to the ED with abdominal pain.^[33]^ PCT was elevated (35.6 ng/mL), along with symptoms of paraneoplastic syndrome such as hypercalcemia, polycythemia, and leucocytosis. Blood and urine cultures remained negative, and local bacterial infections were excluded via an indium-111 WBC whole-body scan.^[33]^ Huang et al reported a 67-year-old male with advanced intrahepatic CCA and multiple liver and lung metastases.^[34]^ Serum PCT was increased (18.63 ng/mL), as were CRP (132.43 mg/L) and leukocytes (WBC: 52.8 × 10^9^/L). See Table S3, Supplemental Digital Content, https://links.lww.com/MD/O999, for further details. Septic shock-like serum PCT elevation (>100 ng/mL) without evidence of sepsis or septic shock was reported in one case of primary liver cancer, in a patient with hepatocellular carcinoma and end-stage liver cirrhosis.^[35]^ To our knowledge, this is the first report of a non-septic, non-HCC liver cancer patient with highly elevated sepsis markers, usually observed only in ICU patients with septic shock.

### 3.3. Elevation of additional inflammatory biomarkers

Presepsin/sCD14-ST is considered a highly specific bacterial sepsis marker that is mainly produced and secreted by monocytes after bacterial phagocytosis and intracellular cleavage of CD14. However, elevated sCD14-ST/presepsin levels have been reported in noninfectious inflammatory disorders, such as systemic lupus erythematosus.^[36]^ Furthermore, Ikegame et al recently reported that monocytes and macrophages secrete presepsin after phagocytosis of CD14-positive neutrophils.^[37]^ Thus, the highly elevated sCD14-ST/presepsin levels in our patient may reflect the excessive phagocytosis of CD14-positive blood cells and/or sCD14-expressing apoptotic hepatocytes in the tumor environment. Furthermore, it should be noted that the excretion of PCT and presepsin might be influenced by renal function. Recently, it has been reported that non-septic patients with acute kidney injury have significantly higher presepsin and PCT levels than healthy controls.^[38,39]^ Therefore, it is reasonable to assume that the impaired renal function may contribute, at least partially, to the elevation of both sepsis biomarkers in this case.

In addition, the inflammatory marker IL-6 was elevated above the normal range. This is in line with previous studies in patients with CCA and liver metastasis, but not consistent with the high IL-6 values (>1000 pg/mL) typically observed during systemic inflammation, sepsis, or septic shock.^[26,40,41]^ Overall, inflammation is a key factor in the carcinogenesis of CCA. Over 90% of CCAs are surrounded by a dense desmoplastic tumor environment comprising cancer-associated fibroblasts and macrophages, which express high levels of IL-6, and recent studies suggest that CCA tumor growth is stimulated by autocrine IL-6-mediated signaling.^[2,42–45]^

### 3.4. Why are sepsis biomarkers elevated in CCA?

The retrospective nature of this case report makes it difficult to further narrow down the possible causes. In particular, we could neither address the secretion of PCT and presepsin by the tumor itself nor the biomarker levels before the last chemotherapy. Nevertheless, it is reasonable to assume that several factors may collectively contribute to the high level of PCT and presepsin in patients with advanced primary hepatic adenocarcinoma, without evidence of neuroendocrine or hepatocellular origin. First, the inflammatory tumor microenvironment may strongly stimulate the release of PCT from hepatocytes, liver-resident, and tumor-associated macrophages, as well as the secretion of presepsin, presumably from activated phagocytes.^[2,42–45]^ Second, excessive liver injury and/or tumor necrosis leads to the unregulated release of PCT and sCD14-ST. Third, the impaired renal function may contribute, at least in part, to the accumulation of PCT and presepsin in the circulation.^[38,39]^

### 3.5. Study limitations

The data presented in this study suggest that the elevated sepsis biomarker levels were primarily due to cancer-related causes rather than alternative infectious or inflammatory processes. However, several limitations of our study should be considered: first, although we excluded systemic bacterial infection and severe sepsis based on negative blood and urine cultures, we cannot completely exclude other non-bacterial or local bacterial infections. Second, although cholangitis was excluded by abdominal sonography, we did not assess for other occult extra-abdominal infections using other advanced imaging techniques. Third, due to the retrospective nature of this study, longitudinal biomarker measurements prior to chemotherapy cycles were not available, preventing a clearer understanding of biomarker fluctuations over time. Fourth, due to the limited sample size, we had to limit our retrospective biomarker analysis, excluding additional inflammatory markers such as lactate, which may have provided further insight. For scientific purposes, the following tests could have been considered, to comprehensively exclude alternative non-tumor related causes of sepsis marker elevation: first, a comprehensive workup for infection, including fungal and viral diagnostics (e.g. β-D-glucan, galactomannan or fungal PCR), as well as other PCR-based pathogen detection from blood or tissue samples, which is more sensitive than traditional culture. Second, whole-body positron emission tomography-CT to identify occult localized infections. However, as discussed above, local bacterial or non-bacterial infections do not typically result in elevated “septic shock-like” sepsis biomarker levels.

### 3.6. Conclusion

While cancer patients need to be closely monitored during immunosuppressive chemotherapy –since any systemic bacterial infection or sepsis requires immediate medical intervention – the unnecessary withdrawal of chemotherapy and administration of antibiotics in certain patients with advanced liver cancer may lead to additional side effects and poor therapeutic outcomes. Therefore, sepsis biomarker results should be interpreted with caution in this specific clinical setting, that is, not in isolation and in the context of other clinical findings. Clinicians and laboratory professionals should be aware that sepsis biomarkers such as PCT or sCD14-ST/presepsin can be extremely elevated in patients with primary liver cancer due to causes other than a systemic infection – not only in patients with neuroendocrine or hepatocellular carcinoma but also in those with advanced metastatic CCA.

## Acknowledgments

The authors would like to thank the patient’s family for their willingness to participate in this report. The authors thank Sebastian Alers for providing scientific writing and editorial support, which was funded by DiaSys Diagnostic GmbH (Holzheim, Germany) in line with Good Publication Practice (GPP3) guidelines.

## Author contributions

**Conceptualization:** Bianca Karnuth, Claus Langer, Matthias Grimmler.

**Investigation:** Bianca Karnuth, Almut Brundert, Claus Langer, Thomas Masetto, Maximilian Jüdt.

**Resources:** Christian Müller, Yuriko Stiegler, Hugo Stiegler.

**Supervision:** Christian Müller, Yuriko Stiegler, Christoph Peter, Matthias Grimmler.

**Writing – original draft:** Bianca Karnuth, Almut Brundert, Matthias Grimmler.

**Writing – review & editing:** Bianca Karnuth, Almut Brundert, Claus Langer, Thomas Masetto, Christian Müller, Yuriko Stiegler, Hugo Stiegler, Christoph Peter, Matthias Grimmler.

## Supplementary Material


