# Supplementary material for: Highly elevated sepsis biomarkers in advanced cholangiocarcinoma without sepsis: A case report and literature review
Source: Medicine (Baltimore). 2025 May 23;104(21):e42115. doi: 10.1097/MD.0000000000042115 (PMC12114018; doi:10.1097/MD.0000000000042115)
Supplement: Supplementary file 1 [file medi-104-e42115-s001.pdf]

Table S1. Full blood test results before and after the initiation of chemotherapy on 12th January 2022.

Analyte	Reference range	18/01/22	17/01/22	12/01/22	05/01/22
Na^+ (mmol/L)	136 – 145	141	139	137	138
K+ (mmol/L)	3.5 – 5.1	4.3	4.3	4.6	4.6
Ca^{2+} (mmol/L)	2.15 – 2.55	**2.08 -**	**2.06 -**	**1.97 -**	**2.1 -**
Urea (mg/dL)	18 – 55		55	42	50
Uric acid (mg/dL)	3.4 – 7.0		5.6	6.9	6.6
Creatinine (mg/dL)	0.67 – 1.17	1.12	1.11	1.06	1.12
eGFR (mL/min/1.73)	86 – 107	**73 -**	**73 -**	**78 -**	**73 -**
Protein total (g/L)	64 – 83		**58.9 -**		
Albumin (g/L)	35 – 52		**30.7 -**		
Bilirubin, total (mg/dL)	0.1 – 1.2		1.0	1.1	0.7
LDH (U/mL)	< 250	229	**256 +**	**267 +**	**255 +**
ALP (U/mL)	40 – 130		**243 +**	**314 +**	**328 +**
AST/GOT (U/mL)	< 50		**69 +**	**118 +**	**119 +**
ALT/GPT (U/mL)	< 50		**45**	**62 +**	**70 +**
GGT (U/mL)	< 60		**519 +**	**520 +**	**576 +**
CK (U/mL)	< 190				46
Lipase (U/mL)	< 60		19		
TSH, basal (mU/L)	0.27 – 4.20		3.29		
CRP (mg/L)	< 5	**39 +**	**36 +**	**50 +**	**48 +**
PCT (ng/mL)	< 0.5	**> 100 ++**	**> 100 ++**		

Analyte	Reference range	18/01/22	17/01/22	12/01/22	05/01/22
WBC (10^9/L)	3.9 – 10.9	**2.4** -	4.86		
RBC (cells/pL)	4.44 – 5.61	**3.11** -	**3.34** -		
Hb (g/dL)	13.5 – 16.9	**9.8** -	**10.6** -		
Hematocrit (L/L)	0.40 – 0.49	**0.30** -	**0.32** -		
MCV (fl)	81.9 – 95.5	95.5	94.3		
MCH (pg)	27.0 – 32.3	31.5	31.7		
MCHC (g/dL)	32.4 – 35.0	33.0	33.7		
RDW (%)	12.0 – 13.6	**16.8** +	**16.9** +		
Platelets (10^9/L)	166 – 308	**85** -	**121** -		
Plateletcrit (%)	0.17 – 0.32	**0.10** -	**0.14** -		
MPV (fl)	9.3 – 12.2	11.9	11.7		
PDW (fl)	10.1 – 15.1	14.0	12.7		
CA 19-9 (U/mL)	< 27				**200** +
CEA (ng/mL)	< 3.8				1.9
PT (%)	74 – 120		**64** -	**55** -	**46** -
INR			1.2	1.3	1.5
PTT (sec.)	23.9 – 33.2		25.3	29.9	**33.8** +
D-dimers (ng/mL)	< 500				**5926** ++

eGFR, estimated glomerular filtration rate; LDH, lactate dehydrogenase; ALP, alkaline phosphatase; AST, aspartate aminotransferase; GOT, glutamic oxaloacetic transaminase; ALT, alanine aminotransferase; GPT, glutamic pyruvic transaminase; GGT, gamma-glutamyl transferase; CK, creatine kinase; CRP, C-reactive protein; PCT, procalcitonin; WBC, white blood cells; RBC, red blood cells; Hb, hemoglobin; MCV, mean corpuscular volume; MCH, mean corpuscular hemoglobin; MCHC, mean corpuscular hemoglobin concentration; RDW, red blood cell distribution width; MPV, mean platelet volume; PDW, platelet distribution width; CEA, carcinoembryonic antigen; CA 19-9, carbohydrate antigen 19-9; PT, prothrombin time; PTT, partial thromboplastin time; INR, international normalized ratio.
