# Supplementary material for: Highly elevated sepsis biomarkers in advanced cholangiocarcinoma without sepsis: A case report and literature review
Source: Medicine (Baltimore). 2025 May 23;104(21):e42115. doi: 10.1097/MD.0000000000042115 (PMC12114018; doi:10.1097/MD.0000000000042115)
Supplement: Supplementary file 2 [file medi-104-e42115-s002.pdf]

Table S2. Additional measurement of inflammatory biomarkers and calcitonin (stored serum sample from 18th January 2022).

	**Assay** (Manufacturer)	**Technology**	**Antibody**	**Analyzer**	**Sample volume**	**Reagent Lot**	**Initial measurement**	**Additional measurements** [3]
**Procalcitonin** (PCT)	Procalcitonin FS (DiaSys Diagnostic Systems)	Particle enhanced turbidimetric immuno-assay (PETIA)	polyclonal	Roche Cobas® c702	8.3 µL	30684	> 100 ng/mL [1]	-
				Jeol JCA-BM6010/C	7.5 µL	30684	> 300 ng/mL [2]	-
	Elecsys® BRAHMS PCT (Roche Diagnostics)	Electro-chemiluminescence immunoassay (ECLIA)	monoclonal	Roche Elecsys® e411	30 µL	00552945	-	> 100 ng/mL
	AFIAS PCT (Boditech)	Fluorescence-based lateral flow immunoassay (FIA)	monoclonal	Boditech AFIAS-1	100 µL	PCRLA66F	-	> 100 ng/mL
**Calcitonin**	Elecsys® Calcitonin (Roche Diagnostics)	Electrochemiluminescence immunoassay (ECLIA)	monoclonal	Roche Cobas® 8000	30 µL	55293100	-	20.9 pg/mL
**Presepsin** (sCD14-ST)	Pathfast™ Presepsin [4] (Mitsubishi Chemicals)	Chemiluminescence immunoassay (CLIA)	monoclonal	Mitsubishi Pathfast™	100 µL	1202204438	-	16,517 (± 2,309) pg/mL
**IL-6**	Elecsys® IL-6 (Roche Diagnostics)	Electrochemiluminescence immunoassay (ECLIA)	monoclonal	Roche Cobas® e411	30 µL	00620928	-	27.6 (± 0.9) pg/mL

[1] Undiluted sample exceeded the assay's upper limit of quantification. [2] Samples were diluted with 0.9% NaCl saline solution. [3] Retrospective analysis of stored serum samples. Serum samples were stored at 2-8°C for 3 days and aliquoted for long-term storage at -80°C. [4] Presepsin is a registered trademark by Mochida Pharmaceutical Co., Ltd. (Tokyo, Japan) for the commercial use on goods and services, including diagnostic test reagents and pharmaceutical preparations.
