# Supplementary material for: Highly elevated sepsis biomarkers in advanced cholangiocarcinoma without sepsis: A case report and literature review
Source: Medicine (Baltimore). 2025 May 23;104(21):e42115. doi: 10.1097/MD.0000000000042115 (PMC12114018; doi:10.1097/MD.0000000000042115)
Supplement: Supplementary file 3 [file medi-104-e42115-s003.pdf]

Table S3. Reported cases of primary liver cancer with highly elevated serum PCT levels without evidence of sepsis.

Case	Age	Sex	Cancer	Lesions	History	Tumor PCT expression	Blood PCT elevation	PCT (ng/mL)	CRP (mg/L)	WBC (10^9/L)	Fever (°C)	GOT (U/L)	GGT (U/L)	Reference
**#1**	55	M	iCCA	liver, bone, lung	arterial hypertension, type 2 diabetes, impaired kidney function	n/d	5 days post-chemotherapy	>100	39	2.4	-	69	519	this report
**#2**	80	M	iCCA	liver	CKD stage III, acute kidney injury	n/d	at ED admission	35.6	n/d	15.6	n/d	73	n/d	[1]
**#3**	67	M	iCCA	liver, lung [1]	-	n/d	1 day post liver biopsy [2]	18.6	132.4	52.8	n/d	73	323	[2]
**#4**	65	M	HCC	liver	chronic HBV hepatitis, end-stage cirrhosis	n/d	at hospital admission	>100	18.8	5.2	-	76	n/d	[3]
**#5**	62	M	HCC	liver	-	+ (IHC)	5 days post-admission	51.6	normal	normal	38.3	normal liver function		[4]
**#6**	22	F	FL-HCC	liver	acute bacterial gastroenteritis nine months before	10-fold (qRT-PCR)	at hospital admission [3]	43.0	145	10.7	39.4	66	n/d	[5]
**#7**	25	F	FL-HCC	liver	-	+ (IHC)	at hospital admission	48.3	191	11.4	38.0	404	86	[6]
**#8**	37	M	FL-HCC	liver	-	n/d	at hospital admission [4]	100	53	10.5	-	49	197	[7]

[1] The authors could not exclude a primary lung tumor (ALK-positive NSCLC). [2] Serum PCT was elevated 1 day after liver biopsy; as were CRP and WBC. All inflammatory markers (PCT, CRP, and WBC) normalized after one month of first-line therapy with ensartinib and regression of liver and lung lesions. [3] Serum PCT normalized after tumor resurrection. [4] Blood PCT level dropped dramatically after hepatectomy.

References

1. Meegada S, Eisen R, Coons G, Verma R. Intrahepatic Cholangiocarcinoma Associated with High Procalcitonin, Hypercalcemia, Polycythemia and Leukocytosis. Cureus. 2020;12:e6587. doi:10.7759/cureus.6587.
2. Huang S, Li D, Huang Y, Lu G, Tian Y, Zhong X, et al. An unresectable and metastatic intrahepatic cholangiocarcinoma with EML4-ALK rearrangement achieving partial response after first-line treatment with ensartinib: a case report. Front Oncol. 2023;13:1191646. doi:10.3389/fonc.2023.1191646.
3. Lu J, Chen C-L, Jin J, Chen J, Yu C-B. Continuous elevation of procalcitonin in cirrhosis combined with hepatic carcinoma: a case report. BMC Infect Dis. 2021;21:29. doi:10.1186/s12879-020-05684-2.
4. Zeng J-T, Wang Y, Wang Y, Luo Z-H, Qing Z, Zhang Y, et al. Elevated procalcitonin levels in the absence of infection in procalcitonin-secretin hepatocellular carcinoma: A case report. World J Clin Cases. 2022;10:10811–6. doi:10.12998/wjcc.v10.i29.10811.
5. Brunel V, Cauliez B, Lacaze L, Riachi G, Gargala G, Francois A, et al. Liver mass in a young adult. Lancet. 2011;378:1196. doi:10.1016/S0140-6736(11)61022-2.
6. Matsumoto K, Kikuchi K, Hara A, Tsunashima H, Tsuneyama K, Doi S. Immunohistochemical detection of procalcitonin in fibrolamellar hepatocellular carcinoma. Clin J Gastroenterol. 2021;14:827–30. doi:10.1007/s12328-021-01354-1.
7. Akbulut S, Tuncer A, Ogut Z, Sahin TT, Koc C. High-Level Procalcitonin in Patient with Mixed Fibrolamellar Hepatocellular Carcinoma: A Case Report and Literature Review. J Gastrointest Cancer. 2022;53:1130–4. doi:10.1007/s12029-021-00731-w.
